# Exploring sense of spaciousness in interior settings: Screen-based assessments with eye tracking, and virtual reality evaluations

**DOI:** 10.3389/fpsyg.2024.1473520

**Published:** 2024-11-29

**Authors:** Alp Tural, Elif Tural

**Affiliations:** School of Design, College of Architecture, Arts, & Design, Virginia Tech, Blacksburg, VA, United States

**Keywords:** sense of spaciousness, virtual reality, eye tracking, spatial perception, interiors

## Abstract

This study investigates the perception of spaciousness in interior environments using screen-based assessments with eye tracking, and virtual reality (VR) technologies. The research explores how four key design elements -view access, view content, materiality, and ceiling geometry- influence perceived spaciousness. Thirty-five college students participated in screen-based and VR-based evaluations of 16 photorealistic interior settings. Eye tracking data were collected during screen-based assessments to analyze visual attention patterns. Statistical analyses included repeated measures ANOVAs, pairwise comparisons, and correlations between screen and VR assessments. Results showed that view access significantly affected perceived spaciousness in both screen and VR conditions, with larger windows correlating to higher spaciousness ratings. Materiality also demonstrated significant effects, with natural and textured materials perceived as more spacious than concrete surfaces. View content and ceiling geometry showed trends towards influencing spaciousness perception but did not reach statistical significance. VR presentations generally yielded higher spaciousness ratings compared to screen-based presentations, suggesting that immersive technologies may enhance spatial perception. Eye tracking analyses revealed common gaze patterns and variations in visual attention across different design conditions. This study contributes to the understanding of how design elements influence spatial perception and demonstrates the potential of integrating eye tracking and VR technologies in environmental psychology research. The findings have implications for evidence-based design practices aimed at enhancing perceived spaciousness in interior environments.

## 1 Introduction

Perceived spaciousness or sense of spaciousness is one of the most significant spatial aspects and affective qualities of the built environment that may influence one’s perception of space. Former research has offered important understanding of elements affecting the perception of spaciousness, with specific factors examined thoroughly. However, the current body of knowledge has limitations. Many investigations have used abstract or simplified settings lacking context, and have not employed photometrically accurate or visually realistic lighting conditions, particularly in virtual reality and computer-generated imagery studies. With the aim of contributing to this area, this study investigates the degree of spaciousness in interiors focusing on key design elements derived from the relevant literature.

Extensive research across the domains of urban design, environmental behavior, architecture, and environmental perception has investigated the affective qualities of built environments and their impact on human subjective experiences. These studies have been informed by several key theoretical frameworks that emphasize experiential and emotional aspects of spatial perception. Phenomenology in architecture has provided a foundation for exploring individuals’ emotional connections with places and spaces, while the Environmental Preference Matrix (Kaplan, 1987) has offered insights into the cognitive and affective processes underlying environmental preferences. Attention Restoration Theory (Kaplan and Kaplan, 1989) has linked spatial qualities to affective states and cognitive system, and the Biophilia Hypothesis (Kellert and Wilson, 1995) has influenced studies on how natural elements in built environments impact emotional responses. These frameworks have informed research within the design disciplines, examining how various elements of architecture and interior design influence human perception and evoke subjective impressions (such as Bower et al., 2019; Banaei et al., 2020; Higuera-Trujillo et al., 2017; Vartanian et al., 2015; Allan et al., 2019).

## 2 Literature on spaciousness

This section examines the key studies on spaciousness perception in interior environments, focusing on boundary design, materiality, room geometry, windows, lighting, and additional mediating variables. These areas form the foundation for our research using eye tracking and virtual reality tools to explore the sense of spaciousness in interior settings.

### 2.1 Boundary design and materiality

Stamps and Krishnan (2006) hypothesized that smoother boundary walls would make the rooms appear more spacious. They studied boundary effects using digitally rendered images of library-like settings with bookshelves and solid cabinet doors for varying surface roughness. Acknowledging several mediating variables, they concluded that boundary roughness did not influence the sense of spaciousness. In a related study, Stamps (2010) examined boundary permeability and perceived spaciousness. While boundary permeability was suggested to be a significant factor, the study’s use of abstract, contextually unrealistic environments (random hulls, Danish megaliths) limits the generalizability of its findings.

Von Castell et al. (2018a, 2018b) studied surface colors (hue and saturation effects on the ceiling and white paint on the periphery) on room size perception. Abstract rooms with varying depths and heights were presented in virtual reality (VR) to assess participants’ sense of spaciousness. They did not find any significant influences on ceiling color in relation to perceived size of the spaces. Although they hypothesized that bounding surface luminance is a determinant of spatial dimension, their findings did not support this statement. Their results might have been influenced by limited luminance adaptation time (5 s for each stimulus) as the surface color variations in the study were achieved by variations of white paint and gray configurations.

Simpson et al. (2018) studied the effect of wallpaper patterns on spaciousness perception in VR. They concluded a stronger correlation between large-scale wallpaper textures and higher degrees of perceived spaciousness. However, the study was limited to diamond shaped gray colored patterns and did not explore whether spaciousness perception was affected by the pattern-dependent luminance contrast ratios or visual complexity.

Wang et al. (2020) focused on wall material texture and spaciousness. They investigated spaciousness in VR using eight different materials and concluded that rooms with textured walls were perceived as less spacious than those without texture. However, they did not find any significant differences in paired comparison of textured surface materials.

### 2.2 Room geometry

Studies exploring spatial geometry and size found horizontal areas to have the strongest effect on sense of spaciousness compared to height (Stamps, 2009; Stamps, 2011). However, only a few experiments were conducted using digitally rendered interior settings. Spaciousness data mostly came from the geometrical analyses of outdoors, including streets and squares. Matusiak and Sudbo (2008) compared the influence of width and height on sense of spaciousness using computer generated images and full-scale settings, noting discrepancies between the simulated and real environments. Also, they found significant differences in the sense of spaciousness between school-aged children and university students, possibly due to the eye height differences of those two diverse age groups. Franz (2005) noted the importance of eye height as an important simulation parameter when collecting spatial perception data.

Isovist research field, which focuses on the mathematical analyses of visible space from specific points, has also contributed to the understanding of spaciousness. Using visibility graphs and view metrics like occlusivity and connectivity, studies explored perceived spaciousness (Ünlü et al., 2022; Franz, 2005).

### 2.3 Windows and lighting

Research on windows and spaciousness indicates a significant relationship between opening size and perceived spaciousness (Imamoglu, 1986; Moscoso et al., 2015). Other studies showed that view area measured in solid angles subtended at the eye position correlates better with sense of spaciousness compared to window area only. Zhao et al., (2019) investigated if view content contributes to sense of spaciousness. They did not find a significant relationship between view content and spaciousness when distance afforded by the openings are greater and when the view content included natural outdoor views.

Inui and Miyata (1973) observed strong correlations between spaciousness and average horizontal illuminance on the working plane and at the rear wall for interior illuminance levels using a model with artificial sky and in a full-scale experimental room. They did not find any interaction between sky luminance and perceived spaciousness, yet it is debatable if the sky luminance in the study was representative as it was limited to three adaptation levels. Flynn et al. (1973, 1979) found that sense of spaciousness was influenced by the intensity, uniformity, and arrangement of lighting. Durak et al. (2007) focused on peripheral illumination and reported that peripheral wall washing technique at high illuminance levels enhanced participants’ sense of spaciousness compared to cove lighting.

The influence of higher light levels on sense of spaciousness was confirmed by Bokharaei and Nasar (2016) and Miyake et al. (2019). However, others demonstrated the context dependency of this influence, finding that darker peripheral walls resulted in a greater sense of spaciousness in a large-scale auditorium space due to dimmer light effecting participants’ judgement of spatial boundary and enclosure (Wänström Lindh et al., 2020).

### 2.4 Additional variables

von Castell et al. (2014) examined how furnishing impacts perceived spaciousness and spatial dimensions of interior spaces using model rooms and virtual reality simulations. In model rooms, furnished spaces were perceived as less spacious but taller compared to unfurnished ones. However, these effects did not translate to virtual reality, where furnishing had no significant impact on perceived spaciousness or dimensions. This might be due to the abstraction of furnishings to cuboids rather than using simplified assets in both data collection sessions. Imamoglu (1986) tested furniture density using a scale model of a conference room and concluded that over furnishing negatively influences sense of spaciousness.

As Franz (2005) and Matusiak and Sudbo (2008) suggested, the interpretation and generalizability of research findings on spaciousness sensation are subject to variability due to perceptual and cognitive differences among diverse demographic groups. These variations may be particularly pronounced when comparing individuals with differing levels of expertise in spatial perception, as well as across different age cohorts, ranging from young children to older adults. Such heterogeneity in spatial cognition and experiential factors underscores the importance of considering participant characteristics in the design and analysis of studies investigating spaciousness sensation in interior environments.

## 3 Research gaps and study objectives

While these studies provide valuable insights into factors influencing spaciousness perception and individual factors have been studied in detail, several gaps in the literature remain. Many studies have relied on rather abstract or simplified environments without context and did not rely on photometrically accurate and visually realistic lighting conditions (specifically, for VR and digitally rendered images). Those factors might have potentially limited their ecological validity. As discussed by Meagher and Marsh (2015), affordance-based approaches might better explain users’ spaciousness perception as those approaches may convey ‘behavioral opportunities offered by the environment’ (p. 782).

Additionally, the use of brief exposure times in some studies may not fully capture the complexity of spatial perception in VR settings. The integration of eye-tracking technology with VR to study spaciousness perception remains underexplored, despite its potential to provide deeper insights into how individuals perceive space as they report their spatial impressions.

Eye-tracking technology has emerged as a valuable tool for understanding human perception and behavior in different contexts and environments, though its application in interior design research is still evolving. Several studies have demonstrated the potential of eye-tracking in examining how people perceive interior, including research by Kwon and Kim (2021), Cho and Suh (2020), Lee et al. (2015), and Suh and Cho (2021). However, the variety of eye-tracking metrics employed across studies has presented challenges in comparing results and gaze behavior, potentially hindering the development of standardized protocols for spatial perception studies. Sussman and Hollander (2021) suggests that eye tracking can better explain how humans visually engage with their environments. Yet, relying solely on common metrics like heatmaps, fixation maps, and number of fixations may not fully capture the complexity of user interaction with the environment.

This study aims to address these gaps by utilizing both eye-tracking and virtual reality technologies to investigate spaciousness perception in photorealistic interior settings. By focusing on four key design elements - view access, view content, materiality and ceiling geometry, it seeks to provide a more comprehensive understanding of how design factors influence the sense of spaciousness in interior environments. Our approach employs a more robust eye-tracking analysis to overcome previous research limitations and better correlate visual behavior with spatial experience.

## 4 Research hypotheses and variables

The study employs a single question survey to assess participants’ perceived sense of spaciousness while visualizing the settings both on screen and in VR simulations. Screen-based eye tracking data collection was implemented for the screen-based stimuli session to investigate potential similarities in participants’ view patterns when assessing environments for degree of spaciousness. Concurrently, VR tools were utilized to explore whether the perceived sense of spaciousness is enhanced in immersive presentations compared to computer screen displays.

*H1: The perceived sense of spaciousness will be significantly higher when environmental stimuli are presented in immersive virtual reality compared to screen-based presentations.*

### 4.1 View access – window size

View access quantifies the amount of window view visible from a user’s location in a space. In this research, the window size was gradually changed to explore if the amount of view area from a set view position effects the sense of spaciousness.

*H2 (SCR;VR): Spaces with larger windows will be evaluated as significantly more spacious compared to spaces with smaller windows or no openings.*

### 4.2 View content

The literature on spaciousness has indicated a need for further research on how the content of a view influences perceived spaciousness from interior spaces. This research explores the relationship between view content and sense of spaciousness, considering four features of nature. The studied views were selected based on environmental preference research and based on the openness and depth they afford from an interior viewpoint.

*H3 (SCR;VR): Particular natural scenes (*e.g.*, views with horizon, open vistas) will demonstrate a stronger positive correlation with perceived sense of spaciousness compared to other natural scenes with boundaries.*

### 4.3 Boundary materials and texture

The study examines the effects of textured vs. smooth wall, floor and ceiling finishes.

*H4 (SCR;VR): Settings with materials that provide texture and sensory richness will be evaluated as significantly more spacious compared to the other settings.*

### 4.4 Ceiling geometry

The geometrical relationships creating spaces have been a topic of interest in environmental preference and aesthetics studies (Stamps, 2013) and more recently in neuroarchitecture (Sussman and Hollander, 2021; Banaei et al., 2020; Vartanian et al., 2015). This study manipulates ceiling design to explore the effect of room geometry on sense of spaciousness.

*H5 (SCR;VR): Rooms with* var*ied ceiling designs (*e.g.*, vaulted, curved, or angled) will be perceived as significantly more spacious compared to the room with flat ceiling*.

## 5 Materials and methods

### 5.1 Participants

The research study included a total of 35 participants, all of whom were enrolled in undergraduate or graduate programs at the college level (Table 1). An *a priori* power analysis was conducted to determine the required sample size for a repeated measures ANOVA with four levels of the within-subjects factor. The analysis was performed using G*Power 3.1 (Faul et al., 2007). We assumed a medium effect size (*f* = 0.25) based on Cohen (1988) guidelines, as no prior studies were available to provide a more precise estimate. Results indicated that a sample size of 24 participants would be required to detect a medium effect size (*f* = 0.25) with 80% power, using an alpha level of 0.05. As this analysis assumed sphericity, we recruited additional participants to account for potential violations of sphericity. The recruitment process was facilitated through the Interior Design program and the Design School email listservs. The gender distribution among the participants was skewed, with females constituting 92% of the sample, mirroring the typical gender distribution within the program cohort from which most of the participants were drawn.

**Table 1:** Distribution of study participants by year of study.

Year of study	*N*	%
Freshman	1	2.9%
Sophomore	5	14.3%
Junior	12	34.3%
Senior	13	37.1%
Graduate+	4	11.4%

The study was conducted under the approval of the University’s Institutional Review Board (IRB 22–322). The consent document for the study was provided along with the digital study signup sheet. In recognition of their participation, each participant was awarded a $20 gift card upon the conclusion of the data collection session, which typically lasted for 30 min.

### 5.2 Procedure

The data collection took place in a faculty office. To prevent daylight from interfering with the eye tracker sensors, and control lighting levels during data collection sessions, window blinds were kept closed. Upon entering the room, participants were briefed on the data collection process and given instructions for both sessions. They provided verbal consent, confirming their voluntary participation. This was followed by eye tracking sensor calibration and the eye tracking data collection session. After completing the eye tacking session, participants were given 2 min to relax their eyes and the PI used the time to prepare the VR/AR session.

To assess screen- and VR-based perceived spaciousness, we employed a self-reported, single-item measure on a 7-point Likert scale. This approach was chosen based on its practicality for repeated measures in VR environments and its demonstrated validity in similar contexts (e.g., Higuera-Trujillo et al., 2017). To capture the overall self-reported spaciousness perception, each participant was asked to answer the following question: ‘please rate the interior setting in terms of the degree of spaciousness’. The use of self-reported measures aligns with established practices in environmental psychology and single-item self-report measures have demonstrated comparable predictive validity in architectural and design research (Kuliga et al., 2021).

### 5.3 Stimulus material and scales

**Figure 1:**
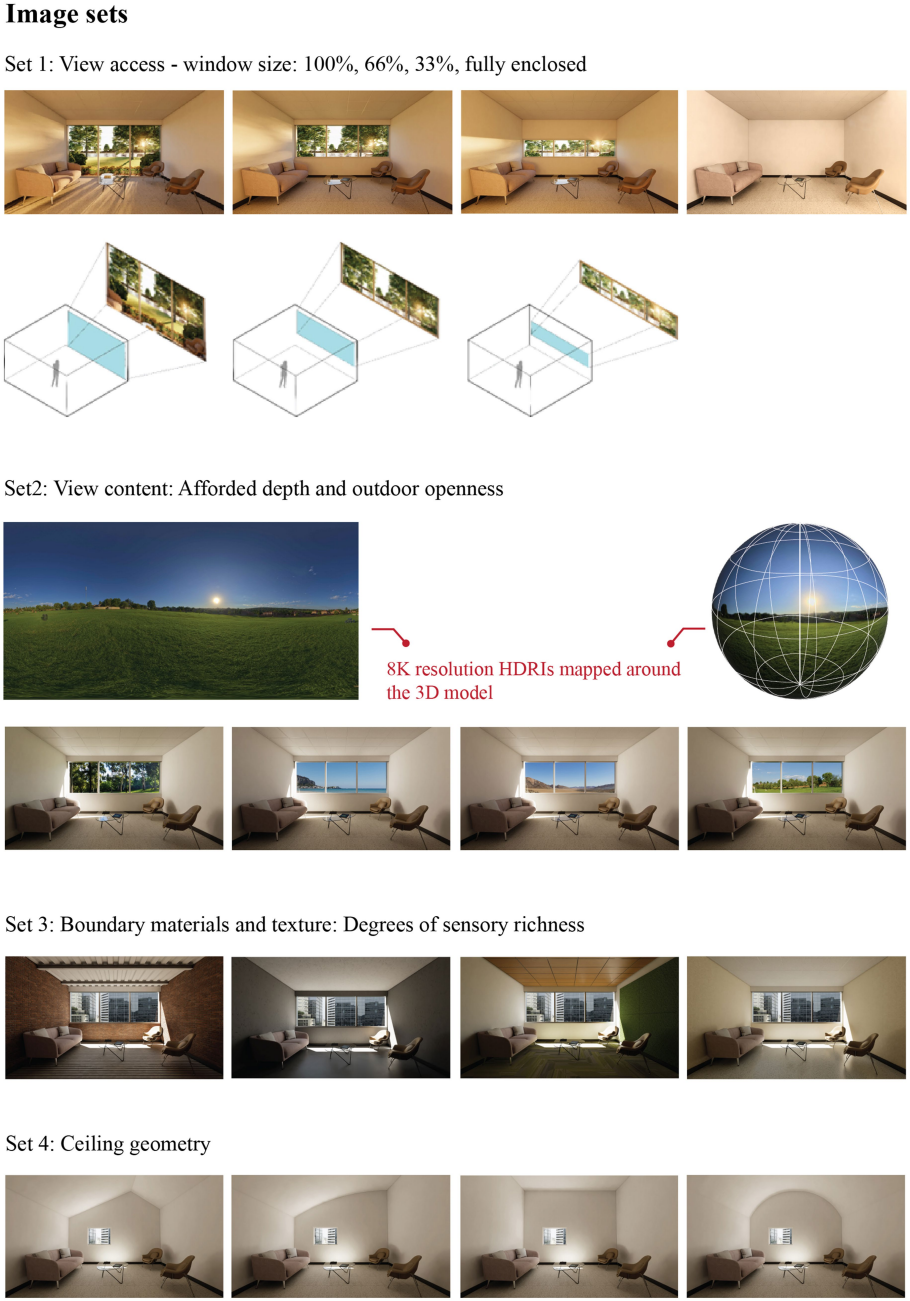
Screen and VR/AR stimuli.

Sixteen different interior settings were modeled using Autodesk Revit software and realistically rendered with the Enscape3D visualization plugin (Figure 1).

For the screen stimuli session, one-point perspective views of each setting were created. Each perspective view was rendered at 2160 by 1,440 pixels matching the display resolution. The camera height for the renders mimicked the average sitting eye height of a 50th percentile US female, positioned at the middle of the back wall. The horizontal view angle of the camera was adjusted to 100 degrees, providing the widest view without parallax based on the depth of the spaces. This setup allowed equivalent coverage of all wall, floor, and ceiling surfaces, with the walls covered approximately 20.5% each, the floor 21%, the front wall 20%, and the ceiling 17.5% of the total pixels in the perspective views. The ceiling plane pixel area within the view was slightly smaller than the other planes, due to the view angle being adjusted five degrees lower than the horizontal sight line to simulate a natural sitting view.

For the VR session of the study, 360-degree panoramic views were created at 8192 by 8,192 pixels using the Enscape panoramic export option. The camera height was adjusted to mimic sitting eye height but was positioned 4 feet away from the back wall, allowing participants to turn around while visualizing the settings in 3D.

All interiors had the same 15 feet by 15 feet footprint. The rooms were designed to resemble a small consultation office, each containing a couch, two chairs, and a coffee table. The ceiling height for the first three sets was consistently 9 feet. For the set with varying ceiling geometries, while maintaining the same floor area, the highest point of the ceilings reached 12 feet, allowing for different ceiling configurations while keeping the overall room volume consistent. All stimuli were carefully controlled for screen luminance, surface colors, and the color spectra of light to ensure consistency across conditions.

The 16 settings were grouped into four subsets:

*Set1: View access and window size:* The degree of view was gradually manipulated from a fully enclosed space to one having a floor-to-ceiling opening located on the wall perpendicular to the view direction (Figure 1). The second and third images in the sequence had openings covering 33 and 66% of the wall area, respectively. The outdoor view was realistically rendered featuring natural elements, such as plants, trees, a lake and filtered natural light. Solar azimuth and altitude angles were set to the same degrees within each set to increase sensory richness and to emphasize change of time.

*Set 2: View content:* Only the view content varied while keeping the opening size, surface finishes and ceiling geometry the same. The four views included a wooded area creating a permeable boundary, a lawn with high degree of extent, a body of water receding into the horizon and a desert environment with shifting topography (Figure 1). To enhance visual fidelity, the view contents were created using 360-degree high dynamic range photographs (HDRI). These images were mapped around the rendered interiors, allowing participants to visualize the views at different angles during the VR session. The HDR images’ natural lighting direction was matched to the scenes’ solar position for consistent lighting distribution among the renders in the set.

*Set 3: Boundary materials and texture*: The view content and opening size remained constant while finish materials for the floors, ceiling and walls were manipulated (Figure 1). Like the second set, the opening size was kept at 66% of the front wall area. The view content for this set was a partial urban downtown 360-degree HDR photograph. The first stimulus featured a setting with brick walls, hardwood floors and steel decking. Concrete was the main material for the second stimulus in this set. The third image included biophilic design features with green wall panels, timber acoustic ceiling tiles and carpet flooring with abstract nature motifs. The last setting had subtle textured wallpaper, and resilient flooring which is a commonly-used type of flexible and durable floor covering such as vinyl, linoleum and rubber.

*Set 4: Ceiling geometry:* The window size was reduced to focus on room geometry and the opening was positioned off-center on the front wall. The window opened to the same urban view used in the second and third sets (Figure 1). All vertical surfaces and the ceiling were finished with matte white paint over gypsum wallboard while the floor material was kept the same with the other three sets as former research findings suggest floor lightness and contrast did not significantly affect spatial perception of rooms as significantly as walls and ceilings (Oberfeld et al., 2010). The highest point for the four ceiling geometries was 10′. The ceiling designs included flat, vaulted, curved, and angled design configurations.

### 5.4 Data collection tools

For the eye tracking session, the perspective renders of the four sets were presented on a Dell 24” QHD screen using Gazepoint Analysis Software. Eye movements were recorded with a Gazepoint GP3 eye tracker operating at a 90 Hz sampling rate. Eye tracking data processing and visualizations were performed using Blickshift Analytics software.

For the VR session of the study, participants were given the option to visualize the 360-degree panoramic renders through either a Meta Quest Pro wireless VR headset or Xreal Air 2 augmented reality (AR) glasses connected to an iPad. This provision aimed to ensure inclusivity and cater to neurodiverse populations and individuals with sensory sensitivities, mitigating adverse effects of VR, such as anxiety due to disconnection from reality, sensory overload and equipment discomfort (Kwon et al., 2023; Dahlstrom-Hakki et al., 2024). The VR headset featured removable partial light blockers on the sides which also allowed the user to adjust immersion level. All participants were shown how to remove these pieces but all VR users chose to keep them attached.

When using the VR headset, participants could visualize the settings through natural head movements. In contrast, AR glasses provided a 130″ screen in front of the participants’ eyes, requiring them to rotate the iPad or interact with the views via the screen to view the settings in 360-degree.

The VR headset was wirelessly connected to a secondary PC through a VR-dedicated router to maintain consistent frame rates, with visualizations managed using Steam Media Player’s panoramic viewer. Meanwhile the AR glasses were connected to an iPad Pro via cable, with panoramic renders accessible through pre-loaded separate tabs on the default browser.

#### 5.4.1 Eye tracking session

Each participant sat approximately 25″ from the screen, with the option to adjust their seating height as needed. The eye tracker sensor provided a digital indicator to ensure optimal distance from the participants’ eyes, with the tracker positioned 1″ to 5″ from the screen, depending on the user’s height and whether they wore makeup, glasses, or lenses. Each participant underwent two eye tracker calibrations before data collection began. They were instructed to remain as still as possible and to examine the images using only their eyes. The eye-tracking session included 18 slides; the first two were informational slides reminding participants about posture and the survey question they would respond to while viewing the images. These informational slides contained only text and were displayed for 25 s each. The remaining stimulus slides were shown for 18 s each, with durations determined through a pilot study (*N* = 6).

#### 5.4.2 VR/AR session

**Figure 2:**
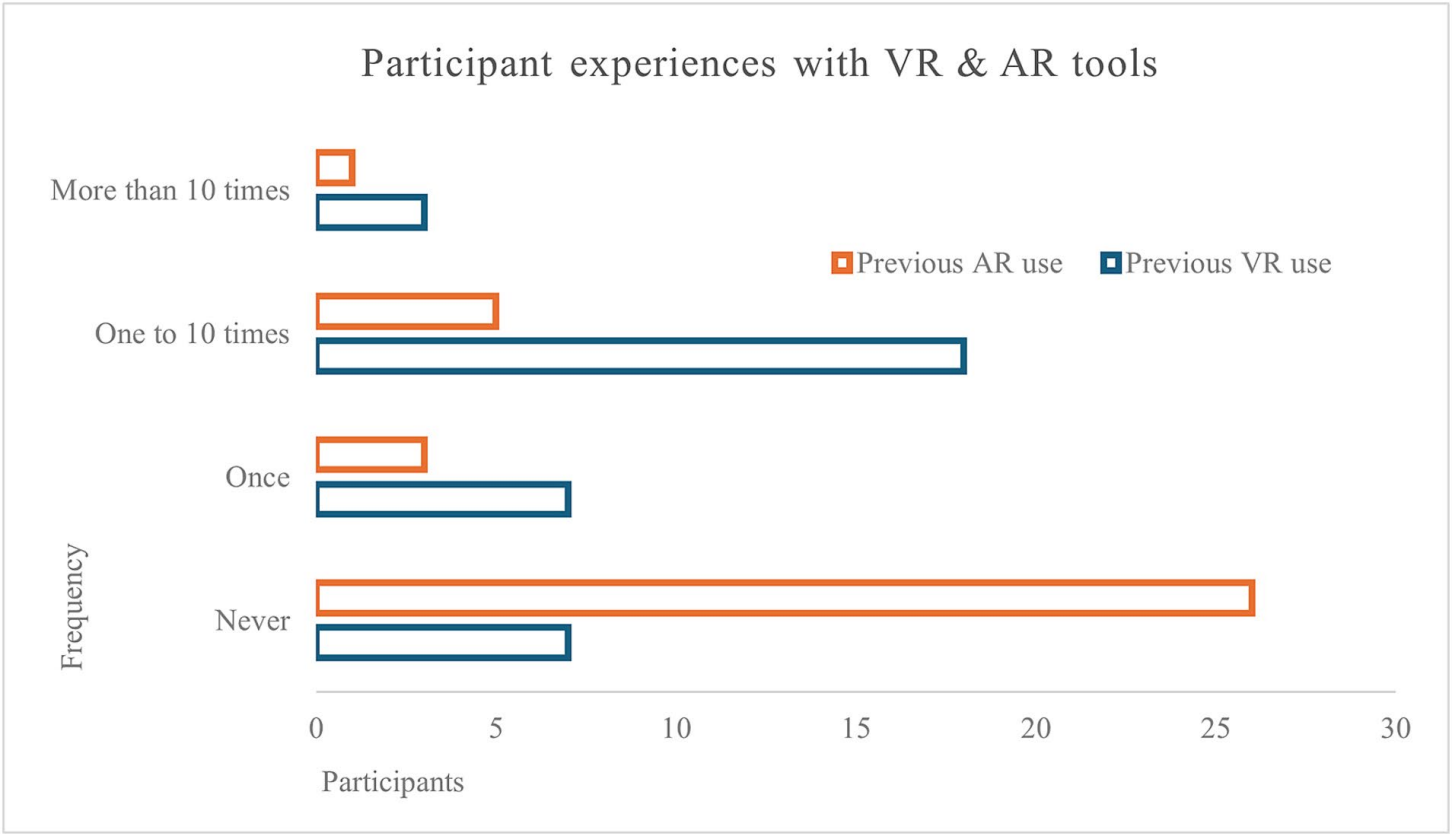
Participants’ experiences with VR and AR tools.

Following the screen session, participants chose either the VR headset or AR glasses for the second session. Using their chosen device, participants could freely look and turn around. Instructions included adjusting VR headset lens spacing to accommodate the user’s interpupillary distance. Unlike the first session, the duration of the stimuli was not limited; participants informed the PI when they were ready to proceed to the next setting. In the study, 1 of the 35 participants opted to use the AR glasses instead of the VR headset. Only one participant mentioned owning a VR headset, the remaining tried using the tools in other classes or for recreation (movies and gaming) (Figure 2).

### 5.5 Eye tracking metrics

**Figure 3:**
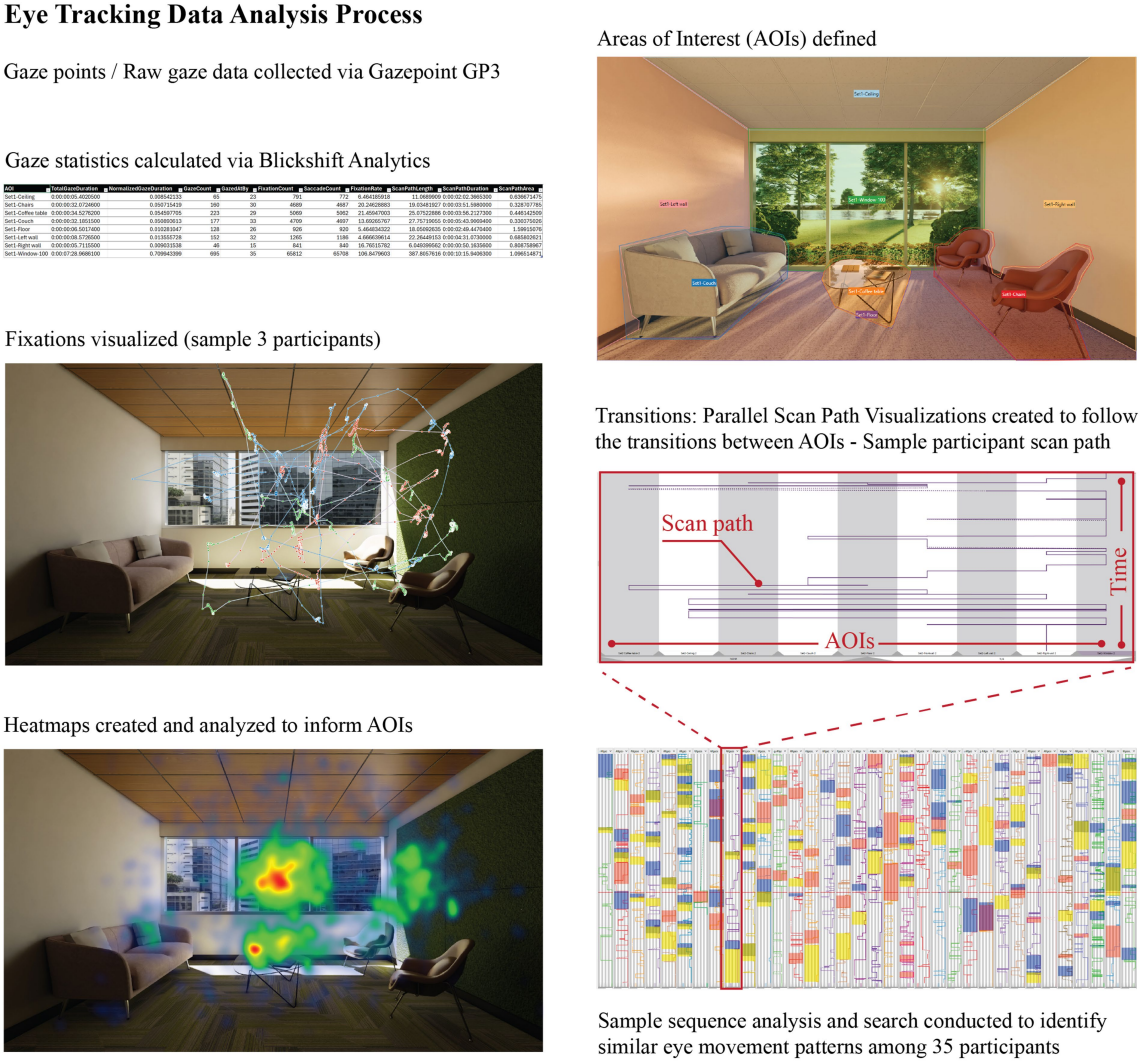
Eye tracking data analyses.

**Figure 4:**
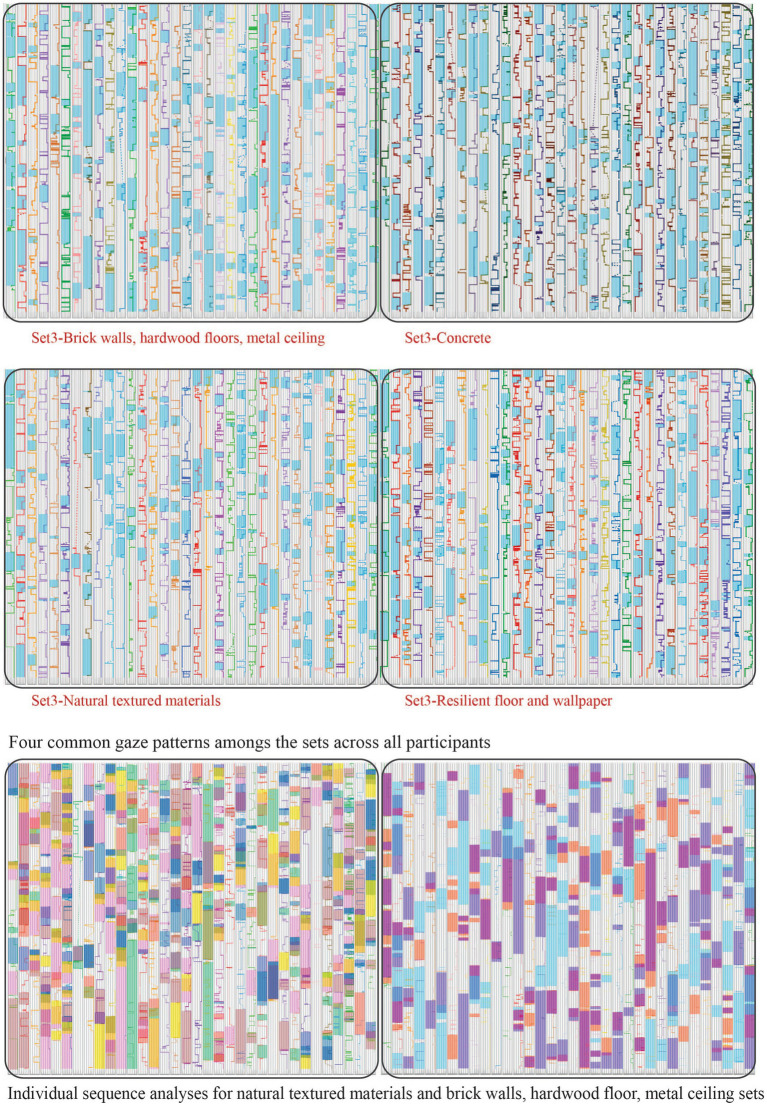
Set 3 - sequence analyses.

In this study, participants’ eye tracking patterns were studied using Blickshift Analytics software, which allowed for a multi-faceted examination of visual behavior. Fixations were derived from raw gaze point data for the 35 participants and those were analyzed in relation to seven areas of interest (AOIs) inclusing floor, wall and ceiling surfaces and furniture. While heatmap visualizations provided a general understanding of attention distribution within selected AOIs, parallel scan paths (PSPs) were specifically employed for examining gaze similarities. PSPs “display the progression of AOIs through time” (Blickshift GMBH, 2024) and allow for gaze sequence analyses to find commonalities amongst participants’ gaze patterns (Figure 3). The color overlays on each scan path column depict a different gaze sequence (such as a participant’s attention moving from the first AOI to the third and then to the second). For each stimulus, the aggregated gaze point data were filtered with a sequence analysis to determine the common gaze patterns amongst all participants. Those sequences were then presented as color overlays on the scan path analysis tables to visualize the common gaze patterns. The sequence analyses were complemented with gaze statistics including the normalized gaze duration for each AOI and total fixation count. This comprehensive method enables a better understanding of how individuals process spatial information and perceive spaciousness, addressing the need for more robust analysis techniques that can better correlate eye-tracking data with subjective perceptions and cognitive processes (Figures 3, 4).

## 6 Results

### 6.1 Comparison of screen-based and virtual reality presentations

*H1: The perceived sense of spaciousness will be significantly higher when environmental stimuli are presented in immersive virtual reality compared to screen-based presentations.*

Table 2 presents the mean perceived spaciousness ratings and correlations between screen and VR presentations for various environmental stimuli. The stimuli were categorized into four sets: View Access, View Content, Boundary Materials and Textures, and Ceiling Geometry. Results indicated that perceived spaciousness ratings varied across different environmental conditions in both screen-based and VR presentations. Pearson correlations between screen and VR assessments were generally moderate to strong and statistically significant (*p* < 0.01) for most conditions (Table 2). The strongest correlation was observed for the vaulted room geometry (*r* = 0.709, *p* < 0.01), while the weakest significant correlation was found for the lawn view content (*r* = 0.157, *p* < 0.01). Notably, VR presentations tended to yield slightly higher mean spaciousness ratings compared to screen-based presentations, particularly for conditions with greater view access and certain room geometries. These findings suggest that while screen and VR assessments of perceived spaciousness are generally consistent, the immersive nature of VR may influence spatial perception in specific environmental contexts in support of the hypothesis.

**Table 2:** Perceived spaciousness ratings and correlations between screen and VR assessments (*N* = 35).

	Screen/Eye Tracking	VR	
	Perceived spaciousness – *M (SD)*	Perceived spaciousness – *M (SD)*	Pearson Correlations – ρ
Set 1: view access			
0%	4.86 (1.167)	4.31 (1.430)	0.415*
33%	4.86 (1.216)	4.97 (1.124)	0.535^**^
66%	5.20 (0.833)	5.66 (0.906)	0.483^**^
100%	5.66 (1.083)	5.91 (1.011)	0.536^**^
Set 2: view content			
Desert	4.89 (1.491)	5.00 (1.414)	0.628**
Water	4.94 (1.187)	5.23 (1.114)	0.500^**^
Woods	4.57 (1.170)	4.63 (1.140)	0.208^**^
Lawn	4.60 (1.006)	4.80 (1.158)	0.157^**^
Set 3: materiality			
Brick	4.37 (1.457)	4.74 (1.291)	0.349**
Concrete	3.46 (1.379)	3.89 (1.568)	0.637^**^
Natural	4.46 (0.980)	4.86 (1.089	0.449^**^
Resilient	4.09 (1.358)	4.37 (1.165)	0.574^**^
Set 4: ceiling geometry			
Curve	5.23 (1.330)	5.03 (1.043)	0.568**
Flat	4.83 (1.248)	5.37 (1.140)	0.604^**^
Angled	4.86 (0.879)	4.91 (1.502)	0.458^**^
Vault	5.23 (1.239)	5.37 (0.973)	0.709**

*p < 0.05 (two-tailed); **p < 0.01 level (two-tailed).

Paired samples *t*-tests revealed significant differences between screen and VR assessments for several environmental conditions (Table 3). These findings suggest that the immersive nature of VR can influence spatial perception differently across various environmental contexts.

**Table 3:** Paired samples *t*-test results comparing screen and VR assessments of environmental stimuli (*N* = 35).

Pair (Screen – VR)	Mean difference	*SD*	*t*	*df*	*p*	Cohen’s d
Set 1: view access						
0%	0.543	1.421	2.26	34	0.03	0.382
33%	−0.114	1.132	−0.597	34	0.554	−0.101
66%	−0.457	0.886	−3.053	34	0.004	−0.516
100%	−0.257	1.01	−1.506	34	0.141	−0.255
Set 2: view content						
Desert	−0.114	1.255	−0.539	34	0.594	−0.091
Water	−0.286	1.152	−1.467	34	0.152	−0.248
Woods	−0.057	1.454	−0.232	34	0.818	−0.039
Lawn	−0.2	1.41	−0.839	34	0.407	−0.142
Set 3: materiality						
Brick	−0.371	1.573	−1.397	34	0.172	−0.236
Concrete	−0.429	1.267	−2.001	34	0.053	−0.338
Natural	−0.4	1.09	−2.171	34	0.037	−0.367
Resilient	−0.286	1.178	−1.435	34	0.16	−0.243
Set 4: room geometry						
Curve	0.2	1.132	1.045	34	0.303	0.177
Flat	−0.543	1.067	−3.011	34	0.005	−0.509
Angled	−0.057	1.349	−0.251	34	0.804	−0.042
Vault	−0.143	0.879	−0.961	34	0.343	−0.162

N = 35. Negative mean differences indicate higher ratings in VR. Cohen’s *d* uses the sample standard deviation of the mean difference.

### 6.2 Effects of view access, view content, materiality and room geometry on perceived spaciousness

One-way general linear model repeated measures analyses of variance (RM ANOVAs) were conducted using SPSS Version 29 to examine the main effect of view access, view content, materiality and room geometry on perceived spaciousness. There were four levels of measurement of each variable. The assumption of normality was verified with normal Q-Q plots. Tables 5, 6 provide the results from the RM ANOVAs for screen- and VR-based evaluations.

**Table 4:** Set 1–100% view access gaze sequence analyses.

Sequence	Pattern 1	Pattern 2	Pattern 3	Pattern 4
1	Window	Window	Window	Window
2	Couch	Coffee Table	Chairs	Left wall
3	Window	Window	Window	Window
				

**Table 5:** Repeated measures ANOVA results for the effect of view access, view content, materiality and room geometry on perceived spaciousness for screen evaluations.

Source	*SS*	*df*	*MS*	*F*	*p*	*η^2^ᵖ*
View access	15.086	3	5.029	6.939	<0.001	0.169
Error	73.914	102	0.725			
View content	3.850	3	1.283	1.821	0.148	0.051
Error	71.900	102	0.705			
Materiality	21.507	3	7.169	5.290	0.002	0.135
Error	138.243	102	1.355			
Room geometry	5.221	3	1.740	2.629	0.054	0.072
Error	67.529	102	0.662			

SS, sum of squares; df, degrees of freedom; MS, mean square; η^2^ᵖ = partial eta squared.

**Table 6:** Repeated measures ANOVA results for the effect of view access, view content, materiality and room geometry on perceived spaciousness for VR.

Source	SS	df	MS	*F*	*p*	η^2^ᵖ	ε
View access	54.429	2.347	23.186	19.363	<0.001	0.363	0.782
Error	95.571	79.814	1.197				
View content	7.029	1.851	3.798	2.970	0.062	0.080	0.617
Error	80.471	62.922	1.279				
Materiality	20.136	3	6.712	7.123	<0.001	0.173	
Error	96.114	102	0.942				
Room geometry	5.829	2.345	2.486	2.765	0.060	0.075	0.782
Error	71.671	79.726	0.899				

SS, sum of squares; df, degrees of freedom; MS, mean square; η^2^ᵖ, partial eta squared; ε, Greenhouse–Geisser correction, if needed.

#### 6.2.1 View access

*H2_screen: Spaces with larger windows will be evaluated as significantly more spacious compared to spaces with smaller windows or no openings.*

Mauchly’s test of sphericity indicated that the assumption of sphericity was not violated for the View Access effect, χ^2^(5) = 6.482, *p* = 0.262. A repeated measures ANOVA revealed a significant main effect of View Access, *F*(3, 102) = 6.939, *p* < 0.001, partial η^2^ = 0.169. Pairwise comparisons using Bonferroni correction showed significant differences between the setting with no windows and the 100% window conditions (*p* < 0.001), as well as between 33 and 100% conditions (*p* = 0.010). The mean scores increased from no opening condition and 33% (*M* = 4.857 for both) to 66% (*M* = 5.200) and 100% (*M* = 5.657).

**Figure 5:**
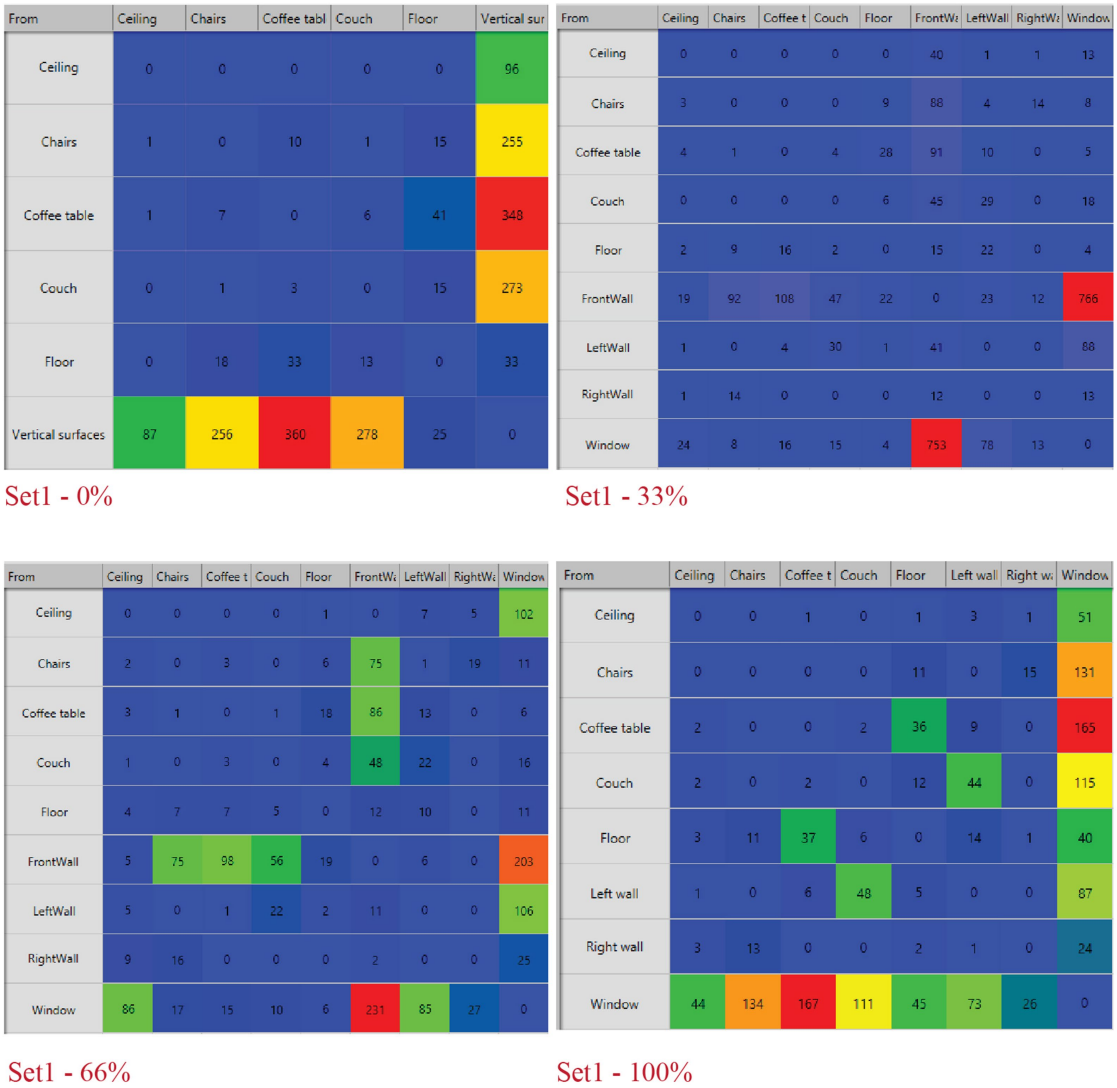
View access -transition matrices.

Eye tracking transition matrices for the view access sets show participants’ gaze transitions between the defined AOIs (Figure 5). Without the window, participants dominantly focused their gaze towards the vertical surfaces, ceiling and furniture. For 33 and 66% window cases the transitions were similar and the gaze sequences were mainly between the window and the surrounding front wall.

**Figure 6:**
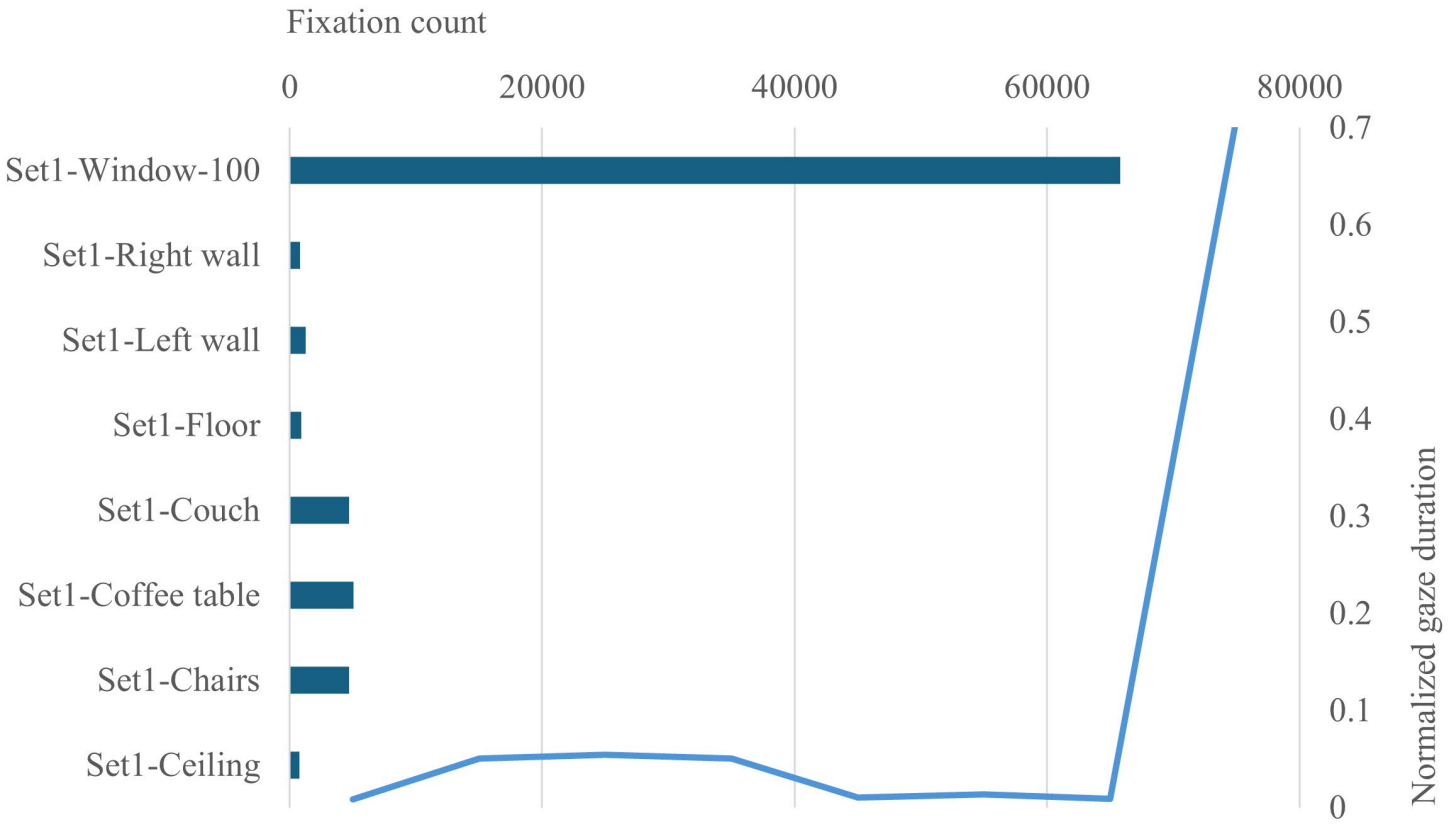
View access −100% window stimuli fixation count and normalized gaze duration per AOI.

**Figure 7:**
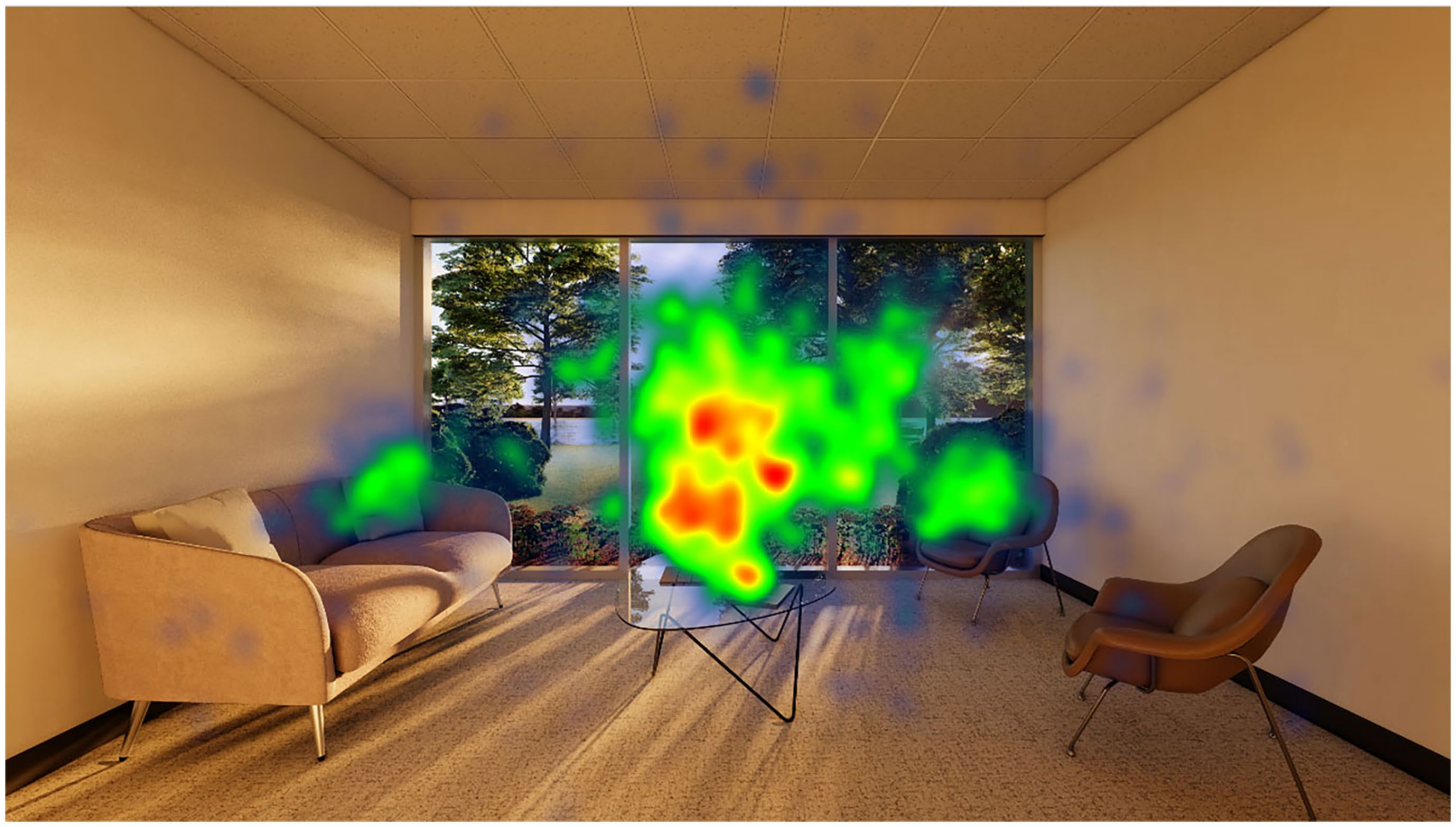
View access – 100% condition heatmap.

Analyses of participants’ eye movements for the 100% opening of the view access set using sequence search revealed four common eye movement patterns. At least 60% of the participants returned to the outdoor view after looking at the furniture (couch, coffee table and chairs) and vertical surfaces during their decision process (Table 4 and Figure 5). This pattern is also confirmed by the fixation count of the window AIO, the total gaze duration and the heatmap (Figures 6, 7).

*H2_VR: Spaces with larger windows will be evaluated as significantly more spacious compared to spaces with smaller windows or no openings.*

Mauchly’s test of sphericity indicated that the assumption of sphericity was violated for the View Access effect, χ^2^(5) = 16.065, *p* = 0.007. Therefore, a Greenhouse–Geisser correction was applied (*ε* = 0.782). A repeated measures ANOVA with a Greenhouse–Geisser correction revealed a significant main effect of View Access, *F*(2.347, 79.814) = 19.363, *p* < 0.001. The effect size was notably larger (partial η^2^ = 0.363), indicating that View Access accounted for 36.3% of the variance in perceived spaciousness when the environments were perceived in VR.

Pairwise comparisons using Bonferroni correction showed significant differences between all View Access levels (*p* < 0.05), except between 66 and 100% opening conditions (*p* = 1.000). The mean differences increased progressively from no opening to 100%, with the largest difference observed between no opening and 100% (MD = 1.600, *p* < 0.001).

These findings support the hypothesis that spaces with larger windows are evaluated as significantly more spacious compared to spaces with smaller windows or no openings, when they are perceived on screen or VR.

#### 6.2.2 View content

*H3_screen: Particular natural scenes (*e.g.*, views with horizon, open vistas) will demonstrate a stronger positive correlation with perceived sense of spaciousness compared to other natural scenes with boundaries.*

Mauchly’s test of sphericity indicated that the assumption of sphericity was not violated for the View Content effect, χ^2^(5) = 4.686, *p* = 0.456. A repeated measures ANOVA revealed no significant main effect of View Content, *F*(3, 102) = 1.821, *p* = 0.148, partial η^2^ = 0.051.

**Figure 8:**
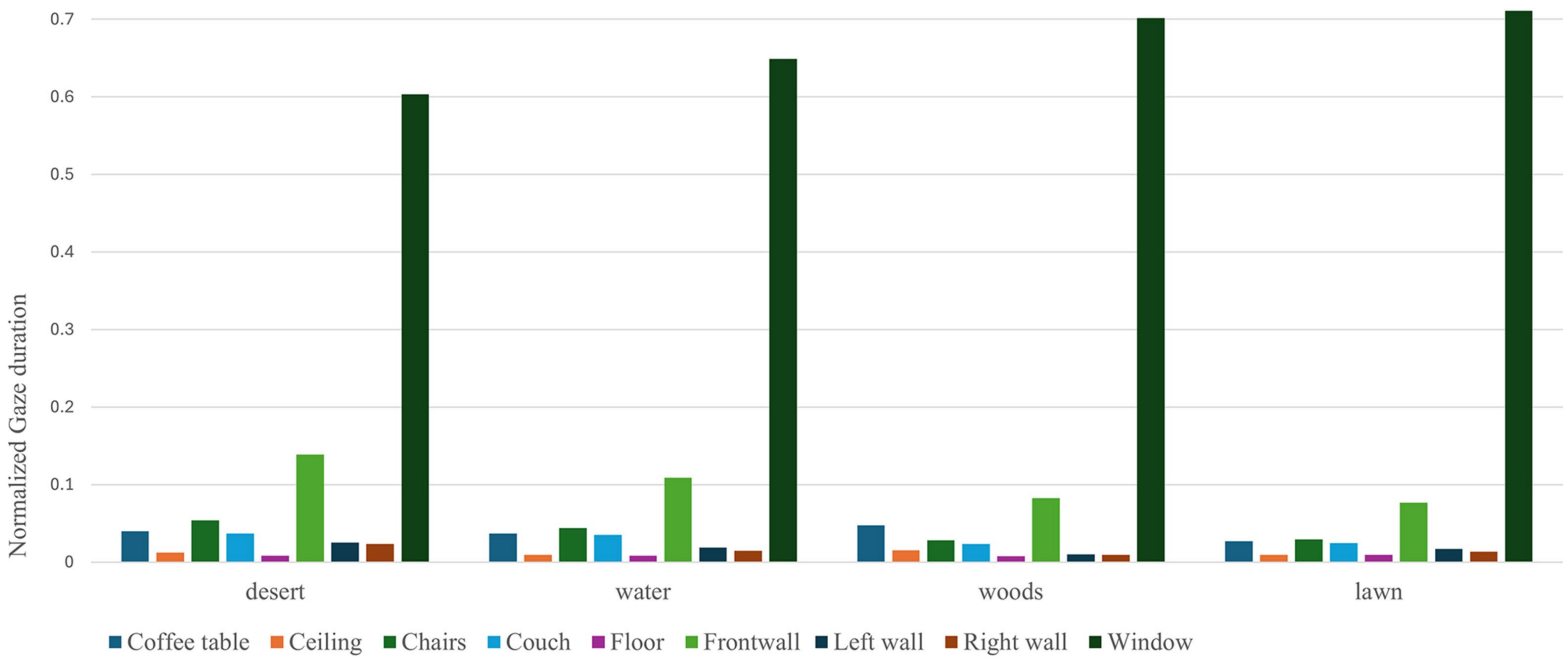
Normalized gaze duration for all AOIs across the view content sets.

Gaze sequence patterns for 100% of the participants in each set were similar with participants looking primarily at the view either fixating their gaze on the surrounding wall first or revisiting it after examining the view content (window-wall-window or wall-window-wall). Other room surfaces and furniture AOIs were not visited as frequently meaning that users reported their sense of spaciousness predominantly looking at the view content (Figure 8).

*H3_VR: Particular natural scenes (*e.g.*, views with horizon, open vistas) will demonstrate a stronger positive correlation with perceived sense of spaciousness compared to other natural scenes with boundaries.*

Mauchly’s test of sphericity indicated that the assumption of sphericity was violated for the View Content effect, χ^2^(5) = 30.019, *p* < 0.001. A repeated measures ANOVA with a Greenhouse–Geisser correction (*ε* = 0.617) revealed a main effect of View Content approaching significance, *F*(1.851, 62.922) = 2.970, *p* = 0.062, partial η^2^ = 0.080.

Despite the overall non-significant effect, pairwise comparisons using Bonferroni correction showed a significant difference between views of water and woods (MD = 0.600, SE = 0.210, *p* = 0.043). No other pairwise comparisons were statistically significant (all *p* > 0.05).

#### 6.2.3 Boundary materials and texture

*H4_screen: Settings with materials that provide texture and sensory richness will be evaluated as significantly more spacious compared to the other settings.*

Mauchly’s test indicated that the assumption of sphericity had not been violated, χ^2^(5) = 9.694, *p* = 0.085. The results showed a significant main effect of materiality on perceived spaciousness, *F*(3, 102) = 5.290, *p* = 0.002, partial η^2^ = 0.135, indicating a medium to large effect size.

*Post hoc* tests using the Bonferroni correction revealed significant differences in perceived spaciousness between several material conditions. The setting with natural materials (*M* = 4.457, SE = 0.166) was rated as significantly more spacious than concrete (*M* = 3.457, SE = 0.233, *p* = 0.004). The room with brick walls, wood flooring and corrugated metal ceiling (*M* = 4.371, SE = 0.246) was also rated as significantly more spacious than concrete finishes (*p* = 0.018). Additionally, the room with resilient finishes (*M* = 4.086, SE = 0.230) was perceived as significantly more spacious than concrete finishes (*p* = 0.035).

These findings partially support the hypothesis. While the setting with natural materials was indeed evaluated as significantly more spacious than one of the other settings (concrete), it was not significantly different from the one with resilient flooring and subtle wallpaper texture. The results suggest that certain material conditions do influence perceived spaciousness, but the relationship may be more complex than initially hypothesized.

Eye tracking sequence analyses comparing all stimuli resulted in three common gaze patterns (Figure 8 top four PSPs). Comparing only the sensory rich pair (natural materials setting and the setting with the brick walls), revealed additional patterns with users visualizing all surfaces except the floor. Each color segment on the PSPs represents a common eye sequence (seven common patterns for the natural setting and three for the one with brick walls) (Figure 4).

Eye tracking descriptive statistics were calculated to compare visual attention patterns across four settings. Brick, concrete, natural, and resilient. Nine Areas of Interest (AOIs) were analyzed: ceiling, chairs, coffee table, couch, floor, front wall, left wall, right wall, and window.

Although the view access and view content variables were controlled, the window AOI received the highest normalized gaze duration (measured in seconds) across all settings (*M*_brick_ = 0.505, *M*_concrete_ = 0.470, *M*_natural_ = 0.383, *M*_resilient_ = 0.458). The window area also had the highest fixation counts (*M*_brick_ = 46,612, *M*_concrete_ = 43,039, *M*_natural_ = 35,251, *M*_resilient_ = 42,129) and gaze counts (*M*_brick_ = 729, *M*_concrete_ = 671, *M*_natural_ = 648, *M*_resilient_ = 704) across all surface types.

In contrast, the floors received the least attention, with the lowest normalized gaze duration (*M*_brick_ = 0.015, *M*_concrete_ = 0.006, *M*_natural_ = 0.023, *M*_resilient_ = 0.020) and fixation counts (*M*_brick_ = 1,331, *M*_concrete_ = 465, *M*_natural_ = 2,146, *M*_resilient_ = 1,777) across all surface types.

Natural surfaces showed the highest variability in gaze patterns for the right wall, receiving substantially more attention (normalized gaze duration = 0.165, fixation count = 15,240) compared to other settings for this AOI. This is notably higher than the values for brick (0.068, 6,260), concrete (0.053, 5,000), and resilient (0.072, 6,723) surfaces.

The setting with the brick walls consistently received high levels of visual attention across most AOIs. They also attracted the most attention for the coffee table (normalized gaze duration = 0.086, fixation count = 7,859) compared to other surface types.

Concrete setting and surfaces showed mixed results, with the highest attention for the chairs AOI (normalized gaze duration = 0.054, fixation count = 4,823).

Natural material settings generally received less visual attention for the window AOI compared to other surface types. However, they attracted more attention to the ceiling (normalized gaze duration = 0.096, fixation count = 8,815) compared to other surface types.

Resilient floor setting surfaces showed moderate levels of attention across most AOIs, often falling between the values of other surface types. They received the highest attention for the left wall (normalized gaze duration = 0.050, fixation count = 4,629) among all surface types although the wall finish was a subtle textured wallpaper.

**Figure 9:**
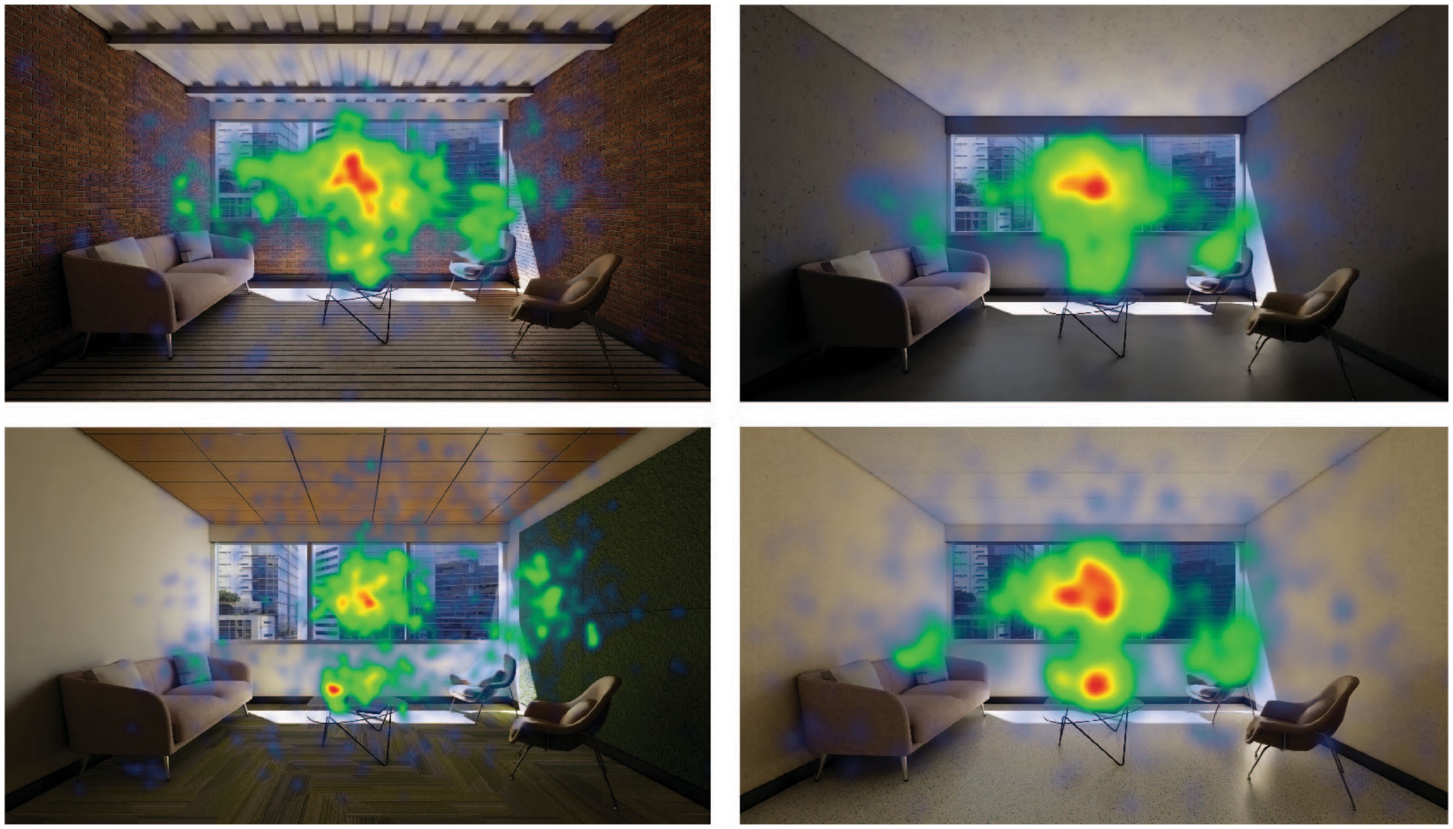
Heatmaps for the boundary materials and texture set.

These descriptive statistics suggest that surface type influences visual attention patterns, with certain AOIs (e.g., window) consistently attracting more attention regardless of the setting, while others (e.g., floor) receive minimal attention. The data also indicate that different surface types may be more visually engaging for specific AOIs, highlighting the complex interaction between surface materials and spatial elements in attracting visual attention. Heatmaps show a slightly wider distribution of fixations for the sensory rich settings (Figure 9).

*H4_VR: Settings with materials that provide texture and sensory richness will be evaluated as significantly more spacious compared to the other settings.*

Mauchly’s test indicated that the assumption of sphericity had not been violated, χ^2^(5) = 5.725, *p* = 0.334. The results showed a significant main effect of materiality on perceived spaciousness, *F*(3, 102) = 7.123, *p* < 0.001, partial η^2^ = 0.173, indicating a large effect size.

*Post hoc* tests using the Bonferroni correction revealed significant differences in perceived spaciousness between several material conditions. The setting with natural materials (*M* = 5.143, SE = 0.180) was rated as significantly more spacious than concrete (*M* = 4.171, SE = 0.226, *p* = 0.005). The setting with brick walls (*M* = 5.029, SE = 0.205) was also rated as significantly more spacious than concrete (*p* = 0.006). There were no significant differences between the setting with resilient finishes (*M* = 4.657, SE = 0.183) and the other settings (all *p* > 0.05).

These findings support the hypothesis. The setting with natural finishes, which presumably provided texture and sensory richness, was indeed evaluated as significantly more spacious than the setting featuring concrete surfaces. However, it was not significantly different from brick or resilient finishes. In support of the hypothesis, the setting with brick walls and wood flooring that provide texture to the room was also perceived as more spacious than concrete.

#### 6.2.4 Ceiling geometry

*H5_screen: Rooms with* var*ied ceiling designs (*e.g.*, vaulted, curved, or angled) will be perceived as significantly more spacious compared to the room with flat ceiling.*

Mauchly’s test indicated that the assumption of sphericity had not been violated, χ^2^(5) = 5.474, *p* = 0.361. The results showed a marginally significant main effect of room geometry on perceived spaciousness, *F*(3, 102) = 2.629, *p* = 0.054, partial η^2^ = 0.072, indicating a medium effect size. This suggests a trend towards differences in perceived spaciousness across room geometries.

*Post hoc* tests using the Bonferroni correction for multiple comparisons revealed no statistically significant differences in perceived spaciousness between any of the room geometry conditions (all *p* > 0.05). The largest mean differences were observed between curved and flat ceilings (MD = 0.400, SE = 0.193, *p* = 0.276), as well as between flat and vaulted ceilings (MD = −0.400, SE = 0.189, *p* = 0.249). However, these differences did not reach statistical significance.

**Figure 10:**
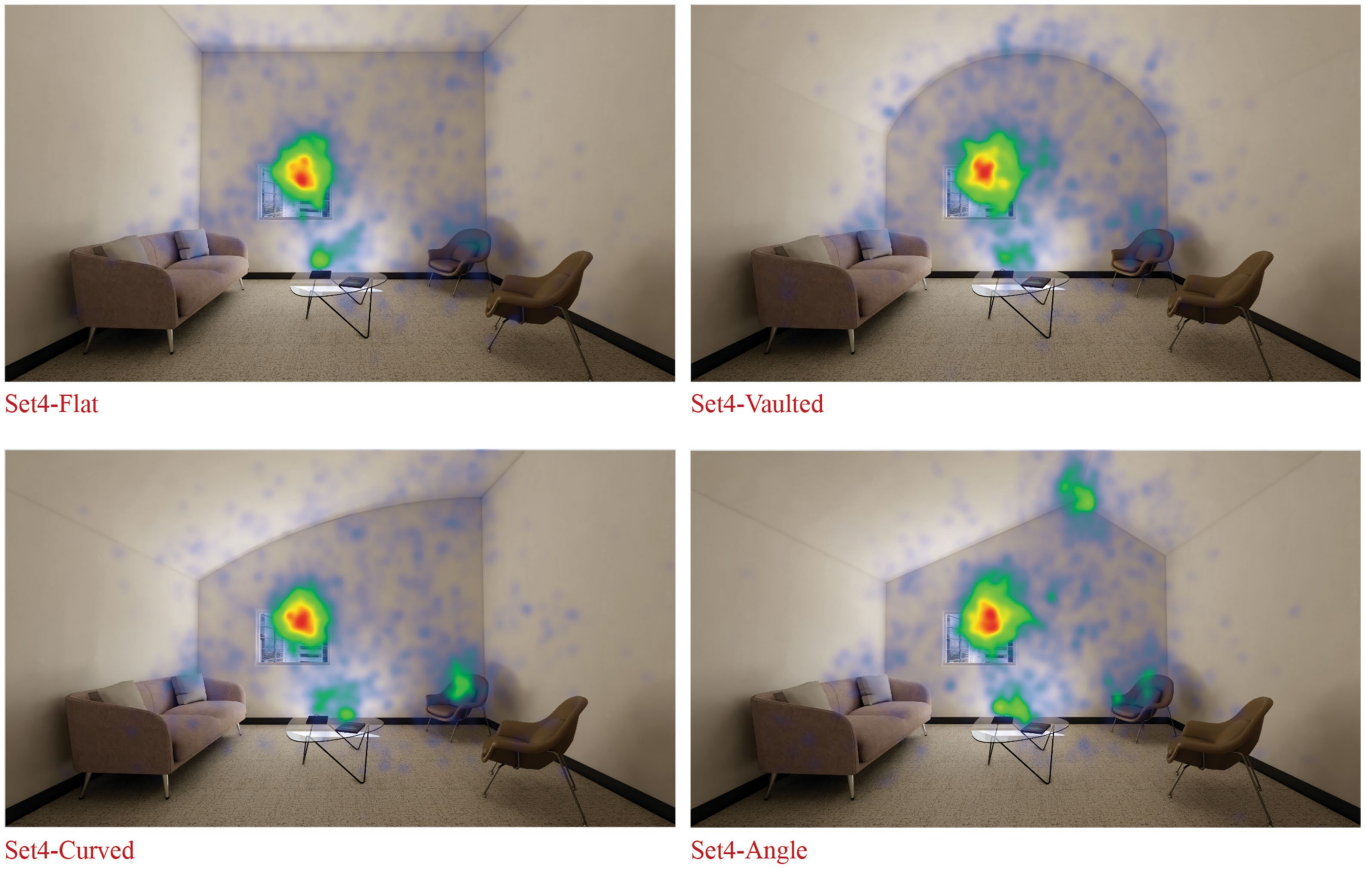
Ceiling geometry – eye tacking heatmaps.

Eye tracking descriptive statistics indicated that participants’ visual attention was predominantly focused on vertical surfaces and ceiling areas when assessing spaciousness across different ceiling geometries. Vertical surfaces consistently received the highest proportion of visual attention (50.1–56.7% of normalized gaze duration) across all ceiling types. Ceilings were the second most attended area, with normalized gaze durations ranging from 16.6 to 25.8%. The angled ceiling attracted the most attention (25.8%) compared to other ceiling types, while the curved ceiling received the least (16.6%). Flat and vaulted ceilings received similar levels of attention (17.2 and 21.6% respectively). Furniture items and floors consistently received less visual attention, while the floor received the least attention across all ceiling types (2.1–3.3%). These findings suggest that when evaluating spaciousness in settings with varying ceiling geometries, participants primarily focused on the vertical surfaces and ceiling areas, with the angled ceiling geometry catching the highest visual attention. Heatmaps indicate that participants’ gaze patterns were concentrated towards the surface edges, which define the ceiling geometry (Figure 10).

*H5_VR: Rooms with varied ceiling designs (*e.g.*, vaulted, curved, or angled) will be perceived as significantly more spacious compared to the room with flat ceiling.*

Mauchly’s test indicated that the assumption of sphericity had been violated, χ^2^(5) = 13.820, *p* = 0.017. Therefore, degrees of freedom were corrected using Greenhouse–Geisser estimates of sphericity (*ε* = 0.782). The results showed a significant main effect of room geometry on perceived spaciousness when assuming sphericity, *F*(3, 102) = 2.765, *p* = 0.046. However, with the Greenhouse–Geisser correction, the effect became marginally significant, *F*(2.345, 79.726) = 2.765, *p* = 0.060, partial η^2^ = 0.075, indicating a medium effect size. This suggests a trend towards differences in perceived spaciousness across room geometries, though it does not reach the conventional threshold for statistical significance with the sphericity correction.

*Post hoc* tests using the Bonferroni correction for multiple comparisons revealed no statistically significant differences in perceived spaciousness between any of the room geometry conditions (all *p* > 0.05). The largest mean differences were observed between the setting with a flat ceiling (*M* = 5.371, SE = 0.193) and the angled ceiling (*M* = 4.914, SE = 0.254), with a mean difference of 0.457 (SE = 0.222, *p* = 0.284). The room with a vaulted ceiling (*M* = 5.371, SE = 0.164) also differed from the angled ceiling by the same magnitude, but this difference was also not statistically significant (*p* = 0.484).

These findings provide limited support for the hypothesis that rooms with varied ceiling designs would be perceived as significantly more spacious compared to rooms with flat ceilings. While there is a trend suggesting potential differences in perceived spaciousness across room geometries, the lack of significant pairwise comparisons and the marginally significant main effect (after sphericity correction) indicate that ceiling design did not have a substantial impact on participants’ perception of spaciousness in this study. Further research with larger sample sizes or different experimental designs might be needed to clarify these potential effects and to determine which specific ceiling designs, if any, contribute to increased perceptions of spaciousness.

## 7 Discussion

This study examined the effects of view access, view content, materiality, and ceiling geometry on perceived spaciousness using both screen-based and virtual reality (VR) presentations, complemented by eye-tracking data. The results provide insights into how these design elements influence spatial perception and highlight the potential benefits of using VR and eye-tracking technologies in interior design research.

### 7.1 Screen-based vs. VR stimuli

The first hypothesis (H1) proposed that sense of spaciousness would be significantly higher in VR presentations compared to screen-based ones. The comparison between screen-based and VR presentations revealed generally consistent ratings across the two modes, with VR tending to yield slightly higher spaciousness ratings. This suggests that while screen-based presentations can provide valuable insights, VR may offer a more immersive and potentially more ecologically valid approach to studying spatial perception.

### 7.2 View access

View Access emerged as a significant factor in perceived spaciousness for both screen-based and VR presentations. Larger windows were consistently associated with higher spaciousness ratings, supporting previous research on the importance of openings in spatial perception (Imamoglu, 1986; Moscoso et al., 2015). The effect was more pronounced in VR, accounting for 36.3% of the variance in perceived spaciousness. This suggests that VR may offer a more sensitive platform for assessing the impact of view access on spatial perception.

Eye-tracking data revealed that participants frequently returned to the outdoor view after examining interior elements, particularly in the 100% window condition. This gaze pattern suggests that larger windows not only increase perceived spaciousness but also may influence how people visually engage with interior spaces, potentially by providing visual relief and a sense of connection to the exterior environment.

### 7.3 View content

The third hypothesis (H3) suggested that particular natural scenes (e.g., views with horizon, open vistas) will demonstrate a stronger positive correlation with perceived sense of spaciousness compared to other natural scenes with boundaries.

The hypothesis was not supported, and view content did not show a significant main effect on perceived spaciousness in either presentation mode. However, a significant difference was found between water and wooded views in VR, indicating that certain natural scenes may influence spatial perception differently. This finding partially aligns with previous research on the impact of view content on spaciousness (Zhao et al., 2019) and suggests that the relationship between view content and perceived spaciousness may be more nuanced than initially hypothesized. In the study, outdoor views were presented using HDRI maps to enhance realism. A more systematic analysis with gradually increasing depth and the outdoor boundary clues could better inform future research.

### 7.4 Boundary materials and texture

The fourth hypothesis (H4) proposed that settings with materials that provide texture and sensory richness will be evaluated as significantly more spacious compared to the other settings. The hypothesis was partially supported, and materiality did not demonstrate a significant effect on perceived spaciousness in both presentation modes unlike former research findings (Wang et al., 2020). Settings with natural materials and brick walls were rated as more spacious than those with concrete surfaces. However, the setting with natural finishes was not evaluated substantially different from the setting with brick walls or the one with resilient floor finish. Eye-tracking data revealed that different materials attracted varying levels of visual attention to specific areas of interest, highlighting the complex interaction between surface materials and spatial elements in shaping visual engagement and spatial perception.

### 7.5 Ceiling geometry

The fifth hypothesis (H5) suggested that settings with varied ceiling designs (e.g., vaulted, curved, or angled) will be perceived as significantly more spacious compared to the setting with flat ceiling. Ceiling geometry showed a marginally significant effect on perceived spaciousness, with trends suggesting potential differences across ceiling designs. The angled ceiling attracted the most visual attention, as indicated by eye-tracking data. While these results do not provide strong support for the hypothesis, they suggest that ceiling design may influence both visual attention and spatial perception in subtle ways that warrant further investigation.

## 8 Conclusion

The integration of eye-tracking technology provided valuable insights into how participants visually engaged with spaces while assessing spaciousness. The observed gaze patterns and attention distribution across different AOIs offer a deeper understanding of the perceptual processes underlying spaciousness judgments, especially for stimuli where spaciousness ratings were not significantly different. This highlights the potential of eye-tracking as a tool for interior design research.

Interestingly, while previous research (Stamps, 2011) identified floor area as a determinant of perceived spaciousness, our eye-tracking analyses revealed that the floor surface was the least visited AOI, regardless of the degree of view access, type of view content, finish materials, and ceiling geometry. This discrepancy underscores the importance of using multiple research methodologies to gain a comprehensive understanding of spaciousness perception.

The study also demonstrates the potential of VR and eye-tracking technologies as tools for both research and design visualization in environment-behavior studies. During the VR data collection sessions, participants had no time limitations due to the unpredictable nature of VR motion sickness. Although no participants reported symptoms suggesting VR-related sickness, future research could benefit from extended exposure times in VR to gather more robust data. Extended VR exposure could impact spatial perception differently from our current findings, potentially revealing adaptation effects or changes in spatial cognition over time. Also, future research can include a comparative study with equal numbers of VR and AR participants to analyze how differences in immersion levels and exposure duration correlate with spatial perception outcomes.

The high proportion of female participants (92%) in our sample, while representative of the program’s demographics, limits the generalizability of our findings on perceived spaciousness to more diverse populations. Future research should aim to incorporate a more balanced gender representation to validate these results across different demographics. Additionally, given the importance of understanding how different immersive technologies might influence spatial perception, future research can include a comparative study with an equal number of participants using VR and AR technologies and an analysis of how differences in immersion levels correlate with spatial perception outcomes.

Pupil dilation has been used as a predictor of cognitive load (Stolte et al., 2020). In our study, participants’ average pupil size statistics were not significantly different between the first and fourth eye-tracking sets. This suggests that the stimuli and task complexity remained consistent throughout the experiment and were not mediating factors in the results. This finding strengthens the internal validity of our data across different stimuli sets. However, some findings might have been influenced by stimuli familiarity and the length of data collection sessions. Future research could address these limitations by comparing screen and wearable eye-tracking tools for collecting interior design-related data or incorporating additional eye-tracking data collection methodologies. For instance, presenting stimuli in groups instead of individually could provide more comparative insights. It is worth noting that the screen-based eye tracker used in this study was limited to a 24-inch screen size, which would result in very small images if the sets were presented in pairs or groups. These findings have significant implications for evidence-based design practices aimed at enhancing perceived spaciousness in interior environments. By combining eye-tracking data with traditional spaciousness ratings, designers and researchers can gain a more nuanced understanding of how people perceive and interact with interior spaces. This integrated approach can lead to more effective design strategies that optimize perceived spaciousness and improve overall user experience in various interior settings.

## Data Availability

The raw data supporting the conclusions of this article will be made available by the authors, without undue reservation.
